# Supplementary material for: Direct nanopatterning of complex 3D surfaces and self-aligned superlattices via molecular-beam holographic lithography
Source: Nat Commun. 2025 Apr 11;16:3436. doi: 10.1038/s41467-025-58651-3 (PMC11986034; doi:10.1038/s41467-025-58651-3)
Supplement: Supplementary file 1 — Supplementary Information [file 41467_2025_58651_MOESM1_ESM.pdf]

Supplementary information

Direct Nanopatterning of Complex 3D Surfaces and Self-Aligned Superlattices via Molecular-Beam Holographic Lithography

Shuangshuang Zeng[§1,2], *Tian Tian*[§3], *Jiwoo Oh*[2], *Zhan-Hong Lin*[2], *Chih-Jen Shih*[2]*

[1] School of Integrated Circuits, Huazhong University of Science and Technology, Wuhan 430074, China

[2] Institute for Chemical and Bioengineering, ETH Zürich, Zürich 8093, Switzerland

[3] Department of Chemical and Materials Engineering, University of Alberta, Alberta T6G1H9, Canada

[§] The authors contributed equally to this work.

[*] To whom correspondence should be addressed: chih-jen.shih@chem.ethz.ch

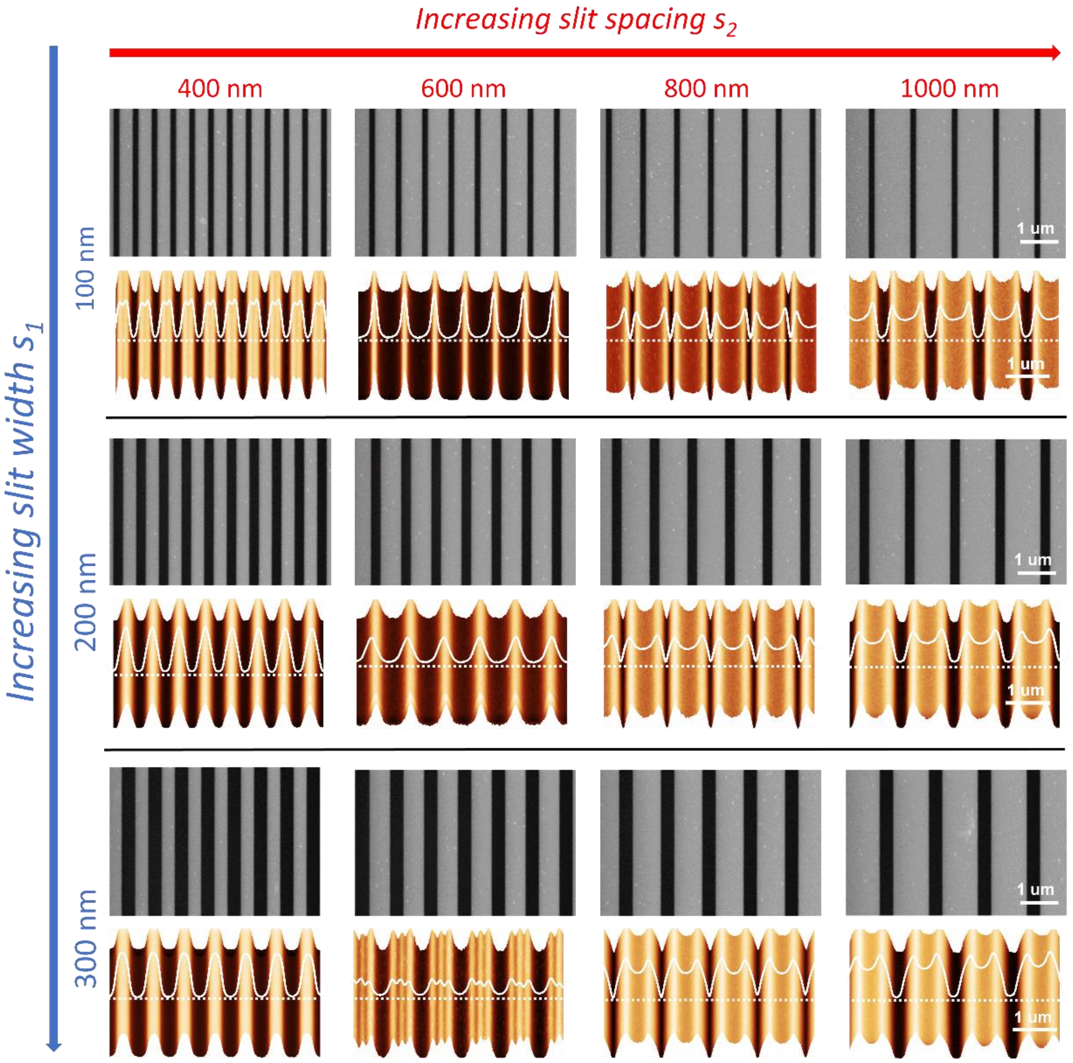

Supplementary Fig. 1. Characterization of the corrugated slit interference patterns. The patterns are formed by evaporating Ge through multiple parallel slits of width s_1 and spacing s_2. The corresponding nanoaperture design for each pattern is also shown.

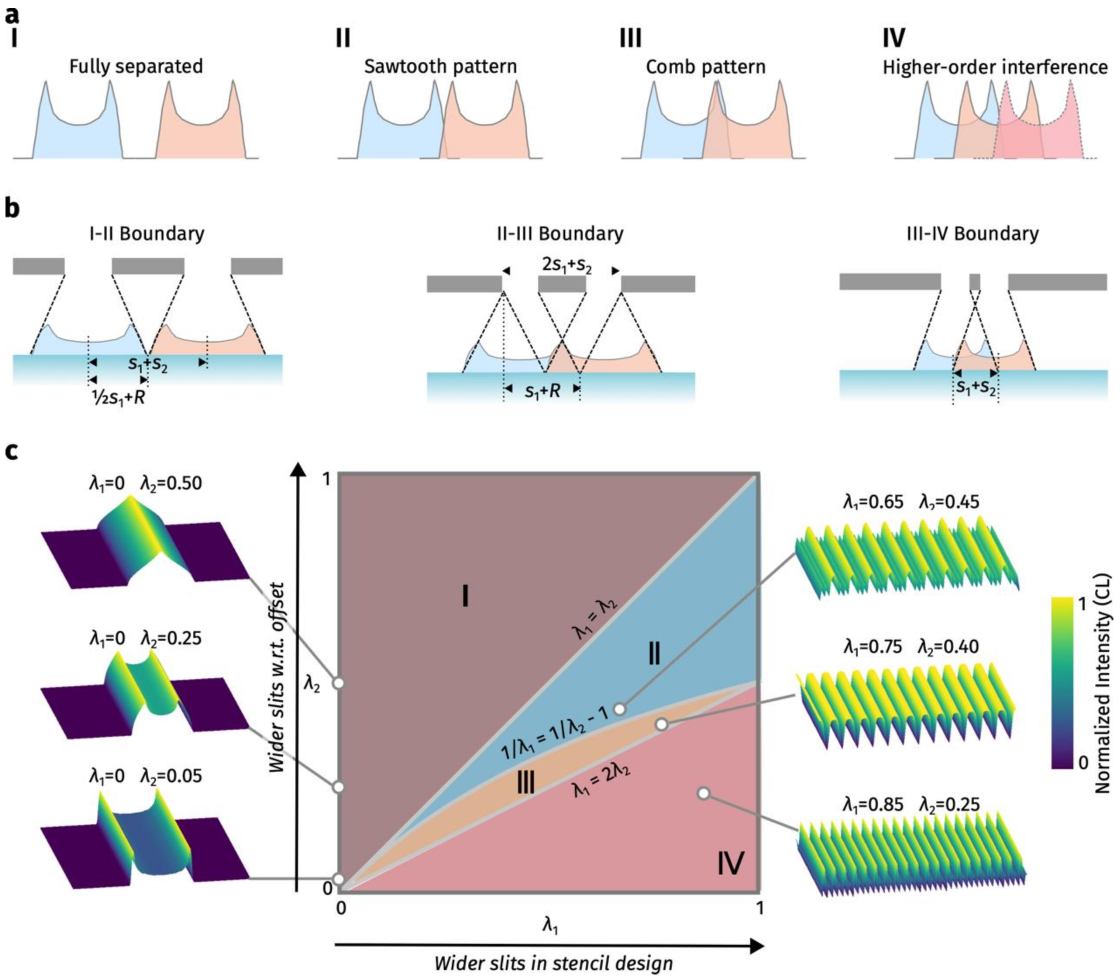

Supplementary Fig. 2. Analysis of one-dimensional interference patterns formed by deposition through nanoslits. a, Schematic representations of the relative positions between the "cathead" deposition patterns from neighboring nanoslits corresponding to the four major regimes in the phase diagram shown in main text Figure 2f. **b**, Schematic representations of the boundary cases between regimes I-II, II-III, and III-IV, respectively. **c**, Examples of CL-simulated MBHL morphology with a wider range of (λ_1, λ_2) parameters. The 3D line profile from isolated nanoslits ($\lambda_1 \rightarrow 0$) becomes sharper with smaller λ_2 values, while the double peak disappears at larger λ_2 values. Source data are provided as a Source Data file.

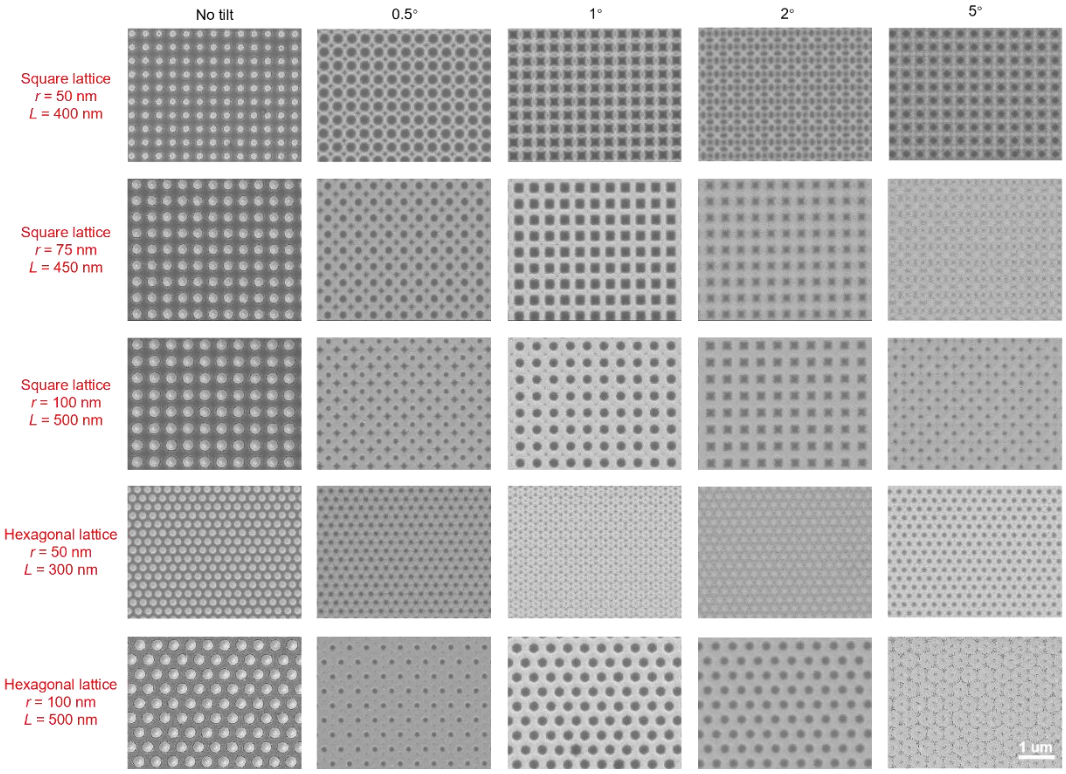

Supplementary Fig. 3. **Characterization of the influence of nanoaperture design and substrate tilting angle on MBHL.** Au is evaporated in various tilting angle and the deposition thickness is 100 nm. The nanoaperture membrane-substrate gap is 2.5μm. Diverse patterns are formed while varying the mask design and substrate tilting angle.

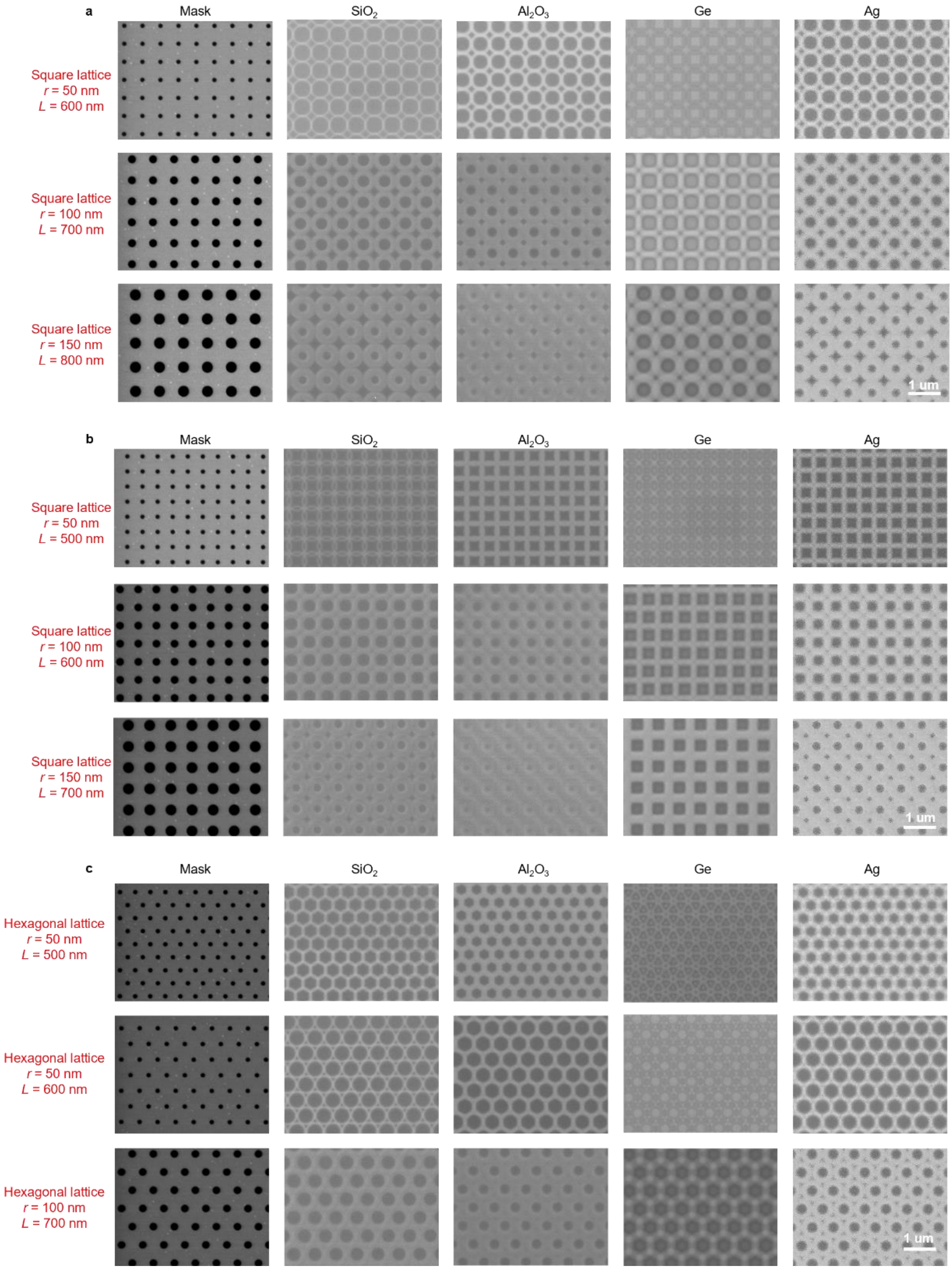

Supplementary Fig. 4. SEM characterization of deposited patterns from various materials through different nanoaperture pattern design. All the materials are evaporated with a thickness of 100 nm and a tilting angle of 5°. The nanoaperture membrane-substrate gap is 2.5μm. **(a, b)** The nanoapertures are arranged in square lattice. Nanoaperture radii vary from 50 nm, 100 nm to 150 nm. The nanoaperture spacing L also changes. **c**, The nanoapertures are arranged in hexagonal lattice. Nanoaperture radii vary from 50 nm to 100 nm. The nanoaperture spacing L also changes.

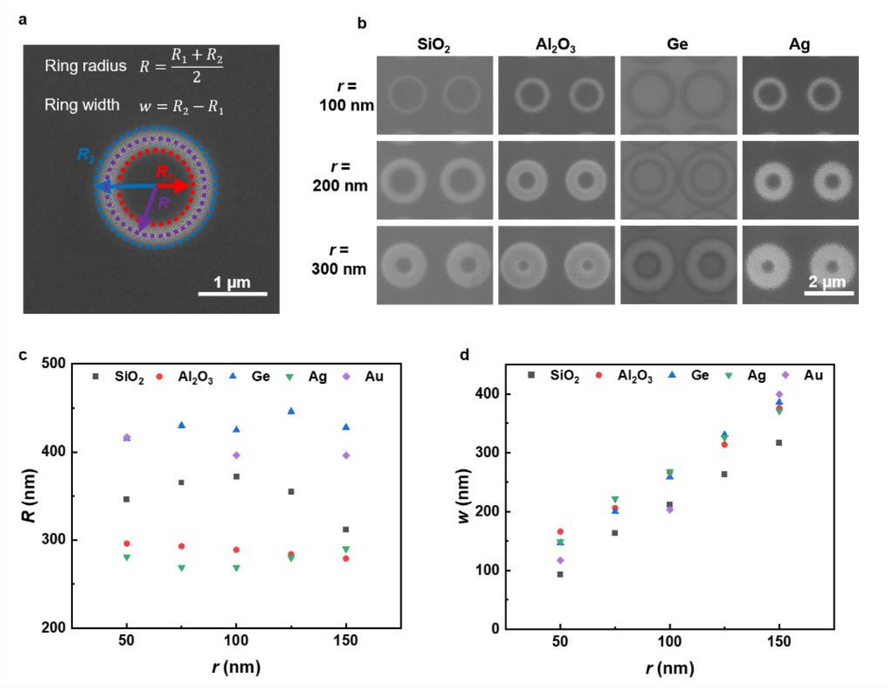

Supplementary Fig. 5. Influence of the material property on deposited ring patterns. a, Deposited rings are fitted by two circles with different radii. The ring radius *R* and ring width *w* are defined as the average and the difference of the radii of the inner and outer circles, respectively. **b**, SEM images of deposited rings from various materials evaporated with a thickness of 100 nm and a tilting angle of 5°. The nanoaperture membrane-substrate gap is 2.5μm. **c**, Dependence of extracted ring radius *R* from different materials on the nanopore radii *r*. **d**, Dependence of extracted ring width *w* from different materials on the nanopore radii *r*.

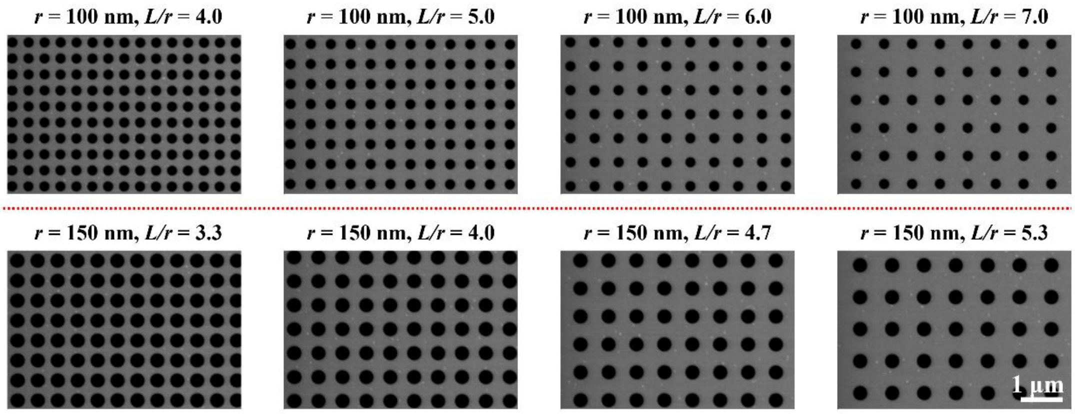

Supplementary Fig. 6. **SEM images of nanoaperture designs for the generated patterns in Figure 4c,d.**

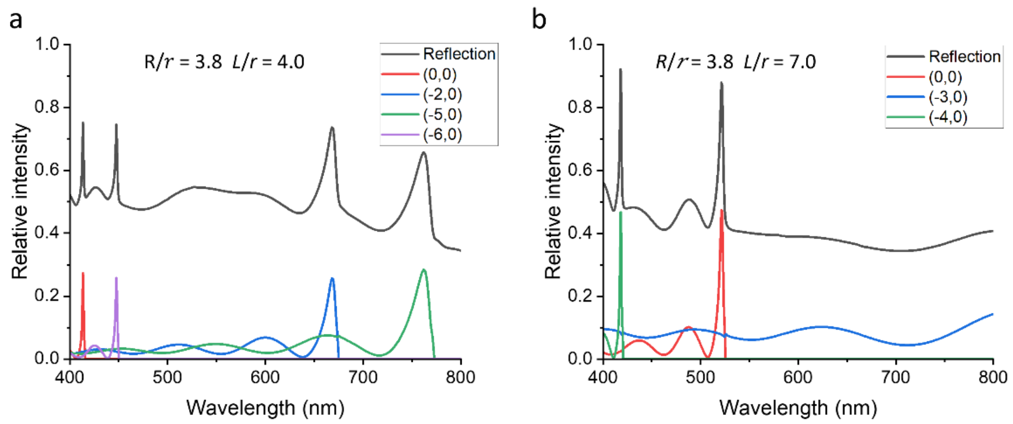

Supplementary Fig. 7. **The reflection spectrum and reflected diffraction orders of the structure in Figure 4c,d. a**, merged lattice structure (*R/r = 3.8; L/r = 4.0*); **b**, waffle structure (*R/r = 3.8; L/r = 7.0*).

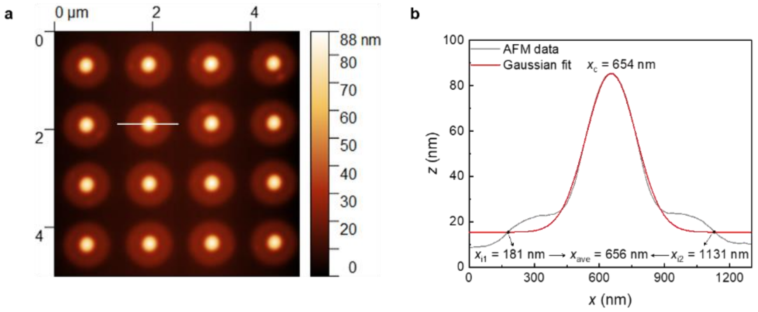

Supplementary Fig. 8. **Determination of the overlay accuracy for the binary nanopatterns. a**, AFM height image of the obtained nanopatterns deposited through nanoapertures of square nanopore lattices, with r=150 nm and L= 1300 nm. **b**, Height profile extracted from the white line in **a**. The white line has been symmetrized to the center of the nanopattern by averaging multiple angular profiles. The overlay accuracy is calculated by measuring the symmetry extent between the central shiny dot and outer ring and is around 2 nm, which is very close to our calculation in the main text. However, this is only a rough estimation owing to the lateral resolution limit from the AFM and it may vary when analyzing different positions.

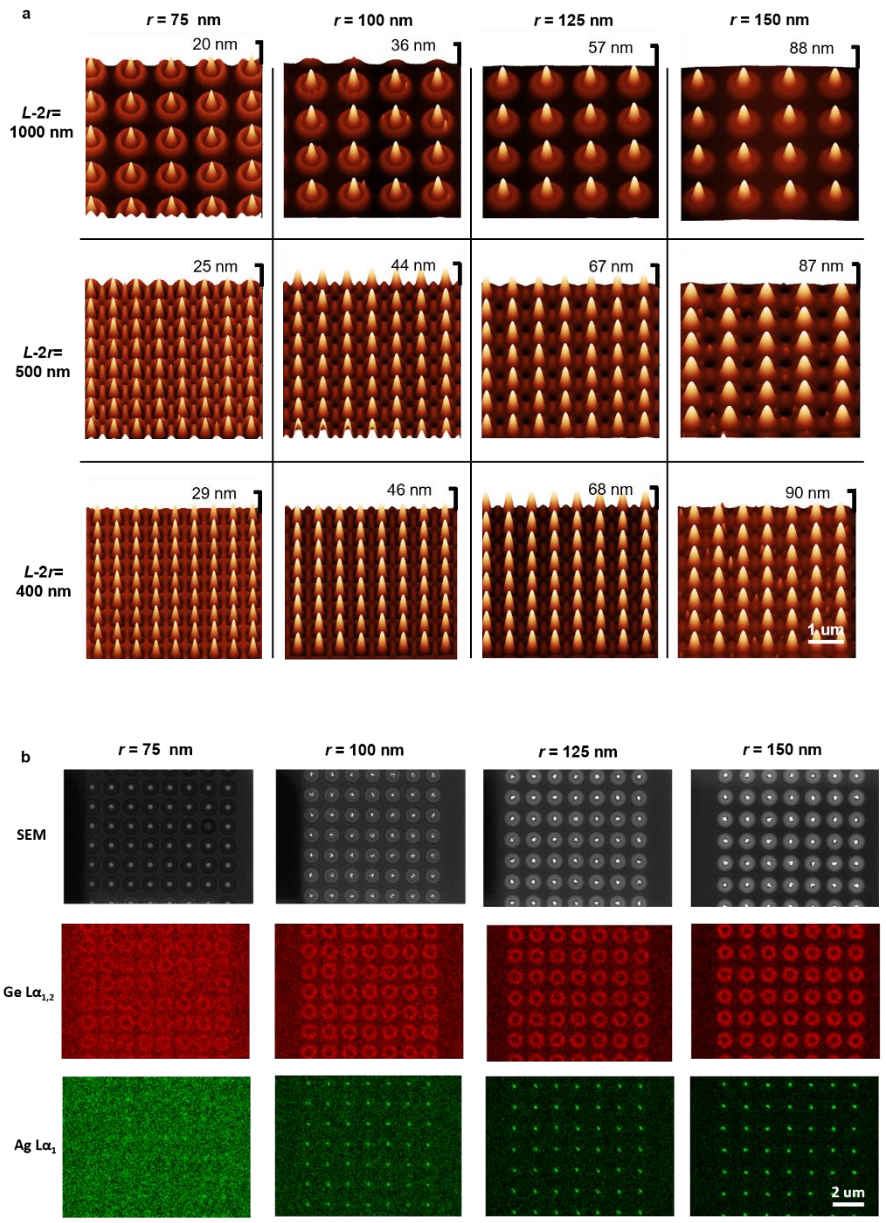

Supplementary Fig. 9. Systematic characterization of deposited binary superlattice pattern combing Ag rings and Ge cones. Ag is evaporated in a tilting angle of 5° and the substrate rotates during the evaporation. Ge is evaporated without tilting. **a**, AFM height images of deposited binary structures by varying the nanoaperture pattern design. The nanopore radii vary from 75 nm to 150 nm with an increment of 25 nm. **b**, EDS mapping of the deposited nanostructures to confirm the existence of different elements.

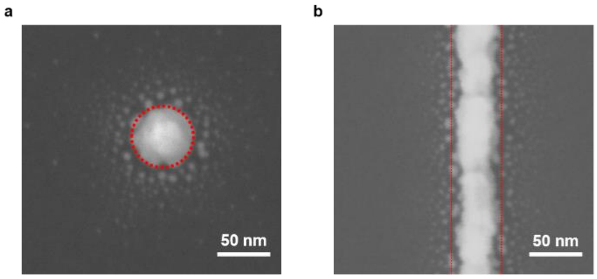

Supplementary Fig. 10. **Critical dimensions achieved using MBHL. a**, Smallest dot structure obtained to be around 57 nm in diameter. **b**, Smallest line structure obtained to be around 47 nm in width. The deposited material is gold with a thickness of 50 nm. The deposition is performed without tilting.

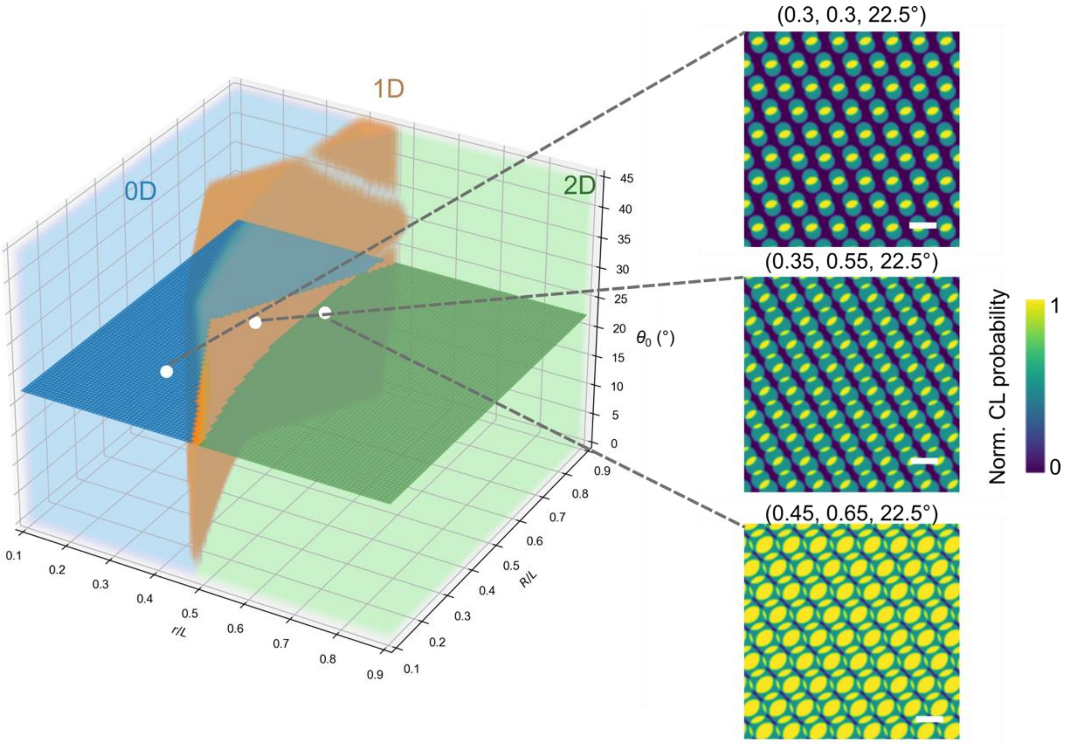

Supplementary Fig. 11. Phase diagram for the dimensionality of MBHL patterns formed by n=2 interference on a hexagonal lattice nanoaperture. The proportion of the 1D (stripe pattern) regime is considerably smaller compared to that of the square lattice nanoaperture shown in main text Figure 6a. The horizontal cut at $\theta_0 = 22.5°$ shows the evolution of pattern geometries, with CL-simulated pattern morphologies for each dimensionality. Scale bars, L (center-to-center distance between nearest apertures). Source data are provided as a Source Data file.

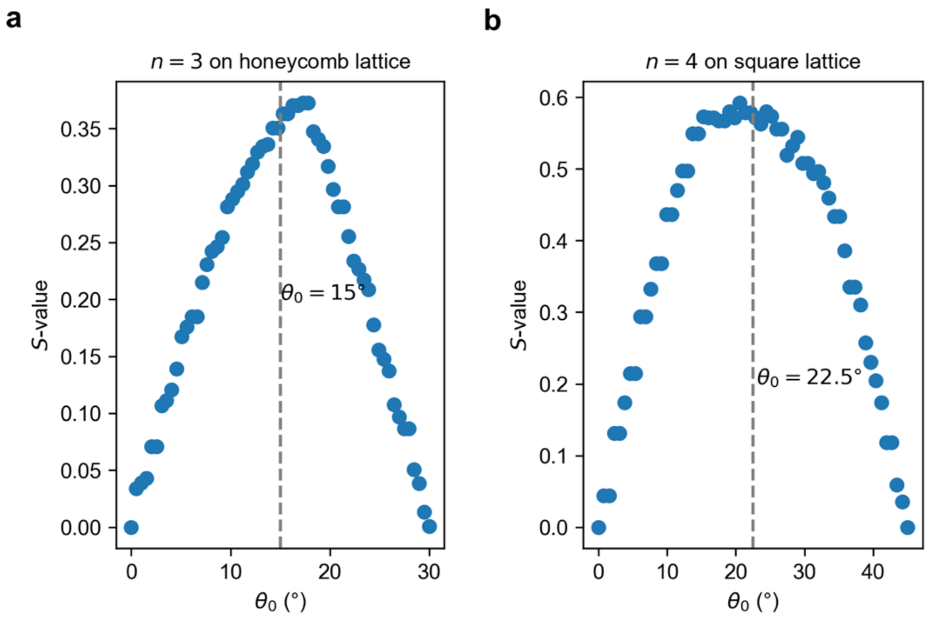

Supplementary Fig. 12. Dependency of the chirality S-value on the angle displacement $\boldsymbol{\theta_0}$. **a,** n=3 interference on square lattice nanoapertures (*r*/*L*=0.45, *R*/*L*=0.55); **b,** n=4 interference (*r*/*L*=0.40, *R*/*L*=0.60) on honeycomb nanoapertures. In both cases, the maximum chirality is achieved when θ_0 is half the value of the angle that the deposition pattern becomes achiral again (30° for honeycomb lattice and 45° for square lattice, respectively). Source data are provided as a Source Data file.

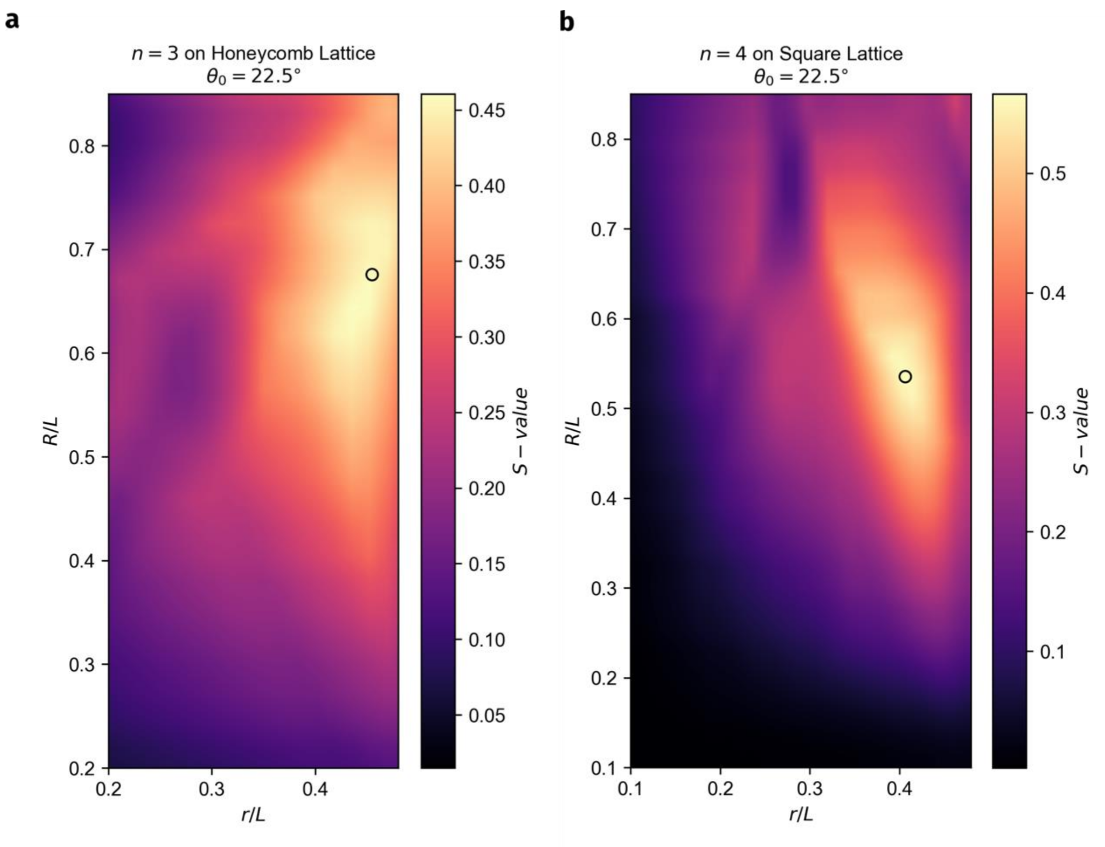

Supplementary Fig. 13. Heatmaps of the chirality index $\boldsymbol{S}$. a, $n = 3$ interference on honeycomb lattice nanoapertures ($\theta_0 = 15°$); **b,** $n = 4$ interference on square nanoapertures ($\theta_0 = 22.5°$) with varied r/L and R/L values. The "hotspots" indicating maximal geometric chirality appear near $(r/L, R/L) = (0.45, 0.68)$ for honeycomb lattice and $(r/L, R/L) = (0.41, 0.54)$ for square lattice, respectively. The corresponding deposition geometries are shown in main text Figure 6b. The black circles indicate the $(r/L, R/L)$ configurations with maximal S-value. Source data are provided as a Source Data file.

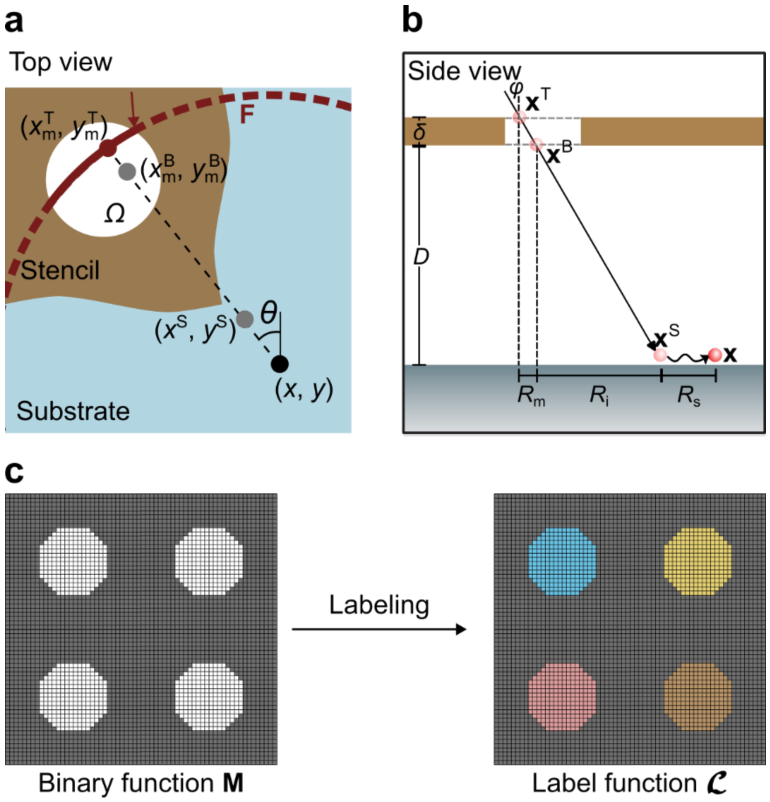

Supplementary Fig. 14. Geometry of the MBHL system considering membrane thickness. a, Schematic of the MBHL system viewed from the top, showing the locations $\mathbf{x}^{\mathrm{T}}, \mathbf{x}^{\mathrm{B}}, \mathbf{x}^{\mathrm{S}}, \mathbf{x}$. The offset trajectory, **F**, is shown as a dark red path. **b**, The same MBHL system viewed from the side. **c**. Conversion of binary function **M** on a regular grid to a label function $\mathcal{L}$.

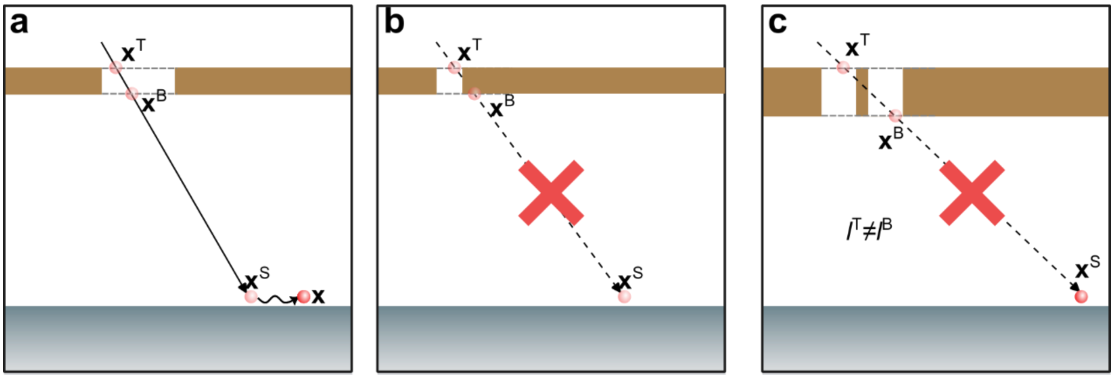

Supplementary Fig. 15. Valid and invalid incident paths when the thickness of the stencil membrane is considered. **a**, A valid incident path, where $\mathbf{x}^{\mathrm{T}}, \mathbf{x}^{\mathrm{B}}$ are both within the same nanoaperture region. **b**, An invalid incident path, where no less than one of $\mathbf{x}^{\mathrm{T}}, \mathbf{x}^{\mathrm{B}}$ are located outside the nanoaperture region. **c**, An invalid incident path, where $\mathbf{x}^{\mathrm{T}}, \mathbf{x}^{\mathrm{B}}$ belong to different hole regions.

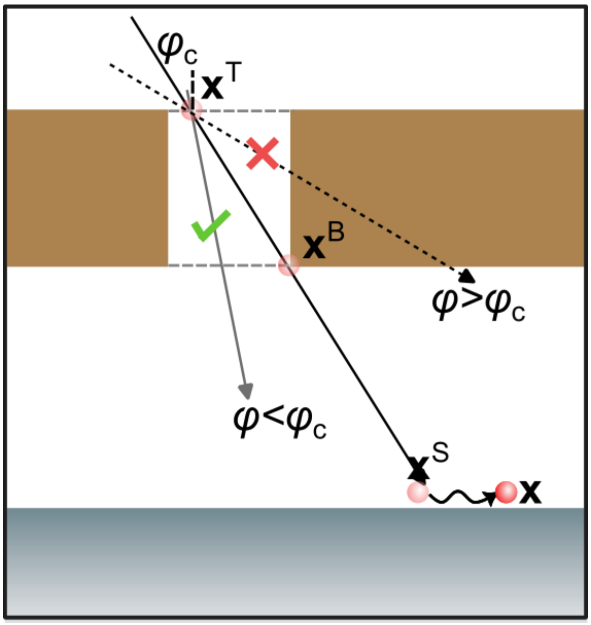

Supplementary Fig. 16. Critical deposition angle φ_c for the stencil membrane. Each incident location $\mathbf{x}^{\mathrm{T}}$ corresponds to a φ_c value so that particles cannot reach the substrate when $\varphi > \varphi_c$. The maximum value for the critical deposition angle, $\hat{\varphi}_c$, corresponds to the value when $\mathbf{x}^{\mathrm{T}}$ lands on the edge of the nanoaperture.

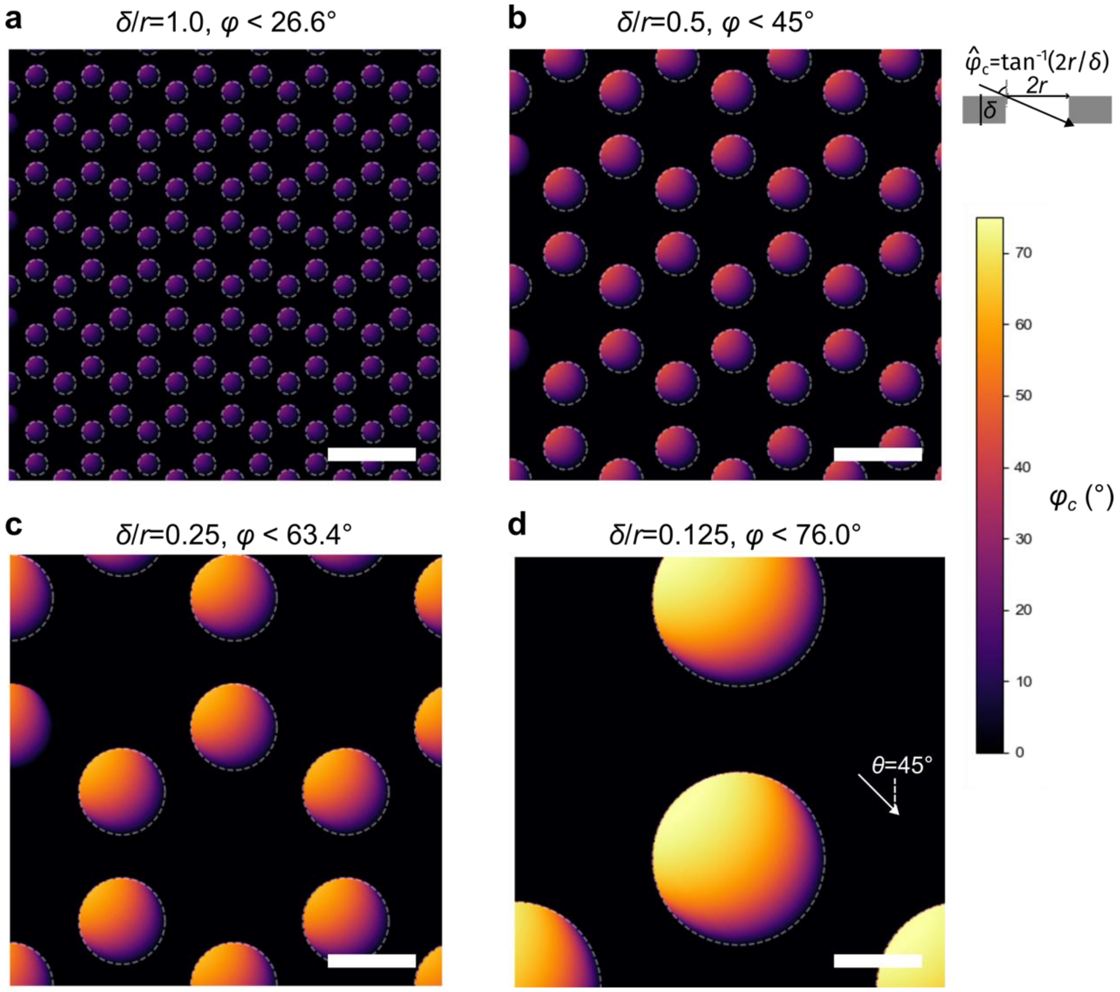

Supplementary Fig. 17. Distribution of critical deposition angle φ_c for the circular nanoapertures on membrane with thickness $\delta = 100$ nm, and incident azimuthal angle $\theta = 45°$. **a**, radius $r = 25$ nm; **b**, radius $r = 50$ nm; **c**, radius $r = 100$ nm; **d**, radius $r = 200$ nm. Scale bars, 200 nm. No particle can reach the substrate surface beyond maximum deposition angle $\hat{\varphi}_c = \tan^{-1} 2r/\delta$. Source data are provided as a Source Data file.

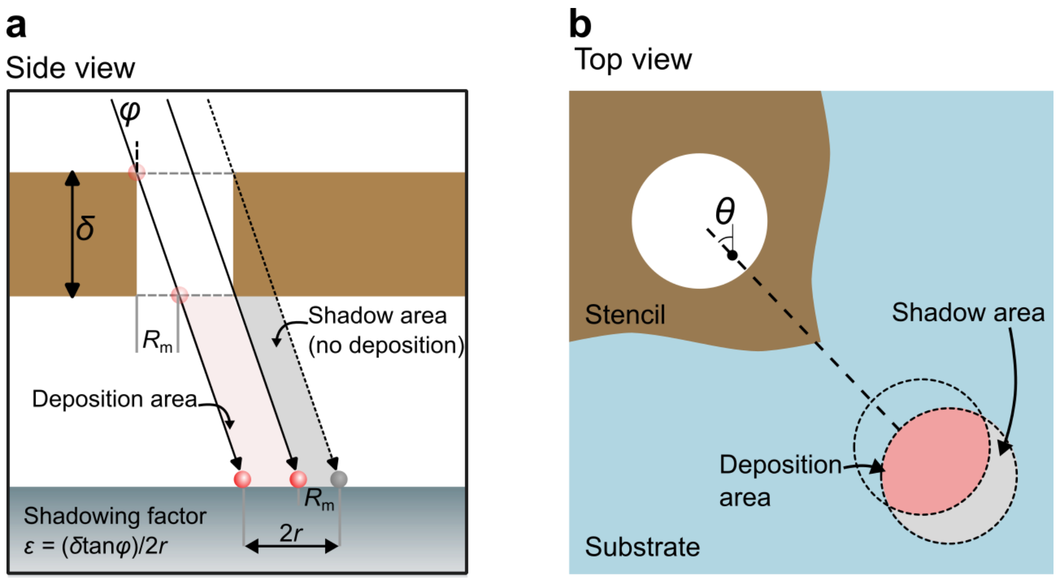

Supplementary Fig. 18. The self-shadowing effect in MBHL. a, Side view of the shadow cast by the stencil wall in high-aspect-ratio nanoapertures. The gray area represents regions where no deposition occurs due to obstruction by the membrane wall. The shadowing factor ε characterizes the proportion of the shadow cast by the membrane wall on the projected pattern. **b,** Top view illustrating the distortion of the deposition pattern caused by the self-shadowing effect. The deposition shape deviates from the ideal nanoaperture pattern due to the increasing influence of ε.

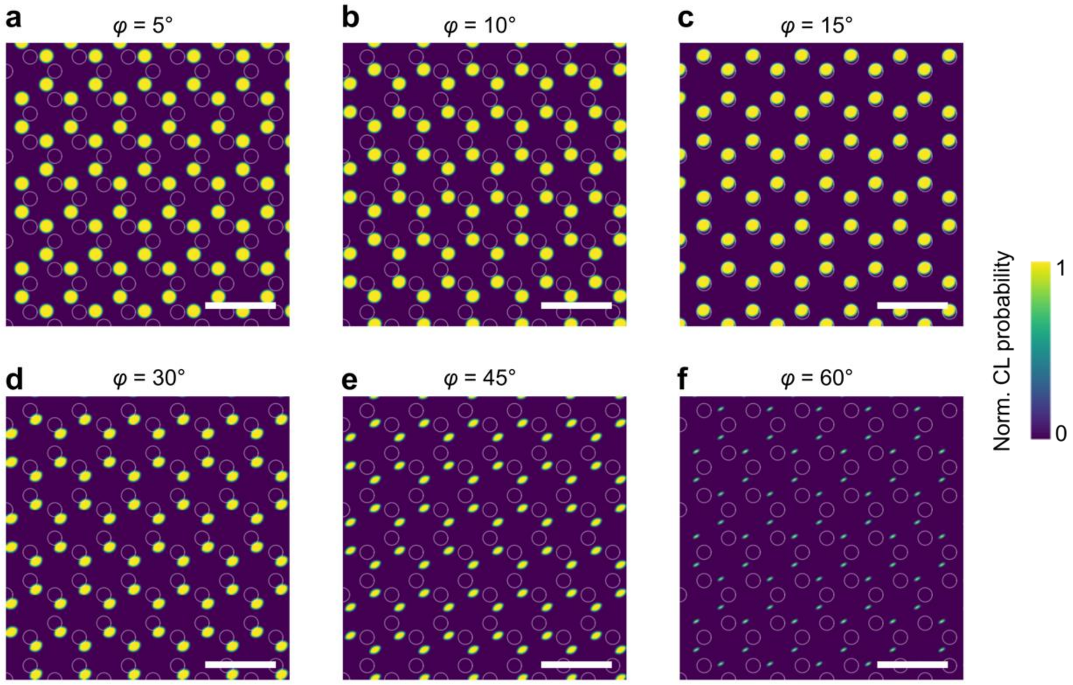

Supplementary Fig. 19. Self-shadowing effect of the stencil membrane in numerical simulations for a honeycomb lattice membrane with $\boldsymbol{\delta = 50}$ nm, nanoaperture radius $\boldsymbol{r = 100}$ nm, and center-to-center spacing $\boldsymbol{L = 400}$ nm under $\boldsymbol{n = 1}$ beam interference ($\boldsymbol{\theta = 30°}$) at different incident angles. a, $\varphi = 5°$; **b,** $\varphi = 10°$; **c,** $\varphi = 15°$; **d,** $\varphi = 30°$; **e,** $\varphi = 45°$ and **f,** $\varphi = 60°$. Scale bars, 500 nm. The locations of the nanoapertures were marked in white circles. The simulations were performed using the raytracing method on periodic stencil lattice. The shadowing effect becomes prominent when $\varphi > 15°$. Source data are provided as a Source Data file.

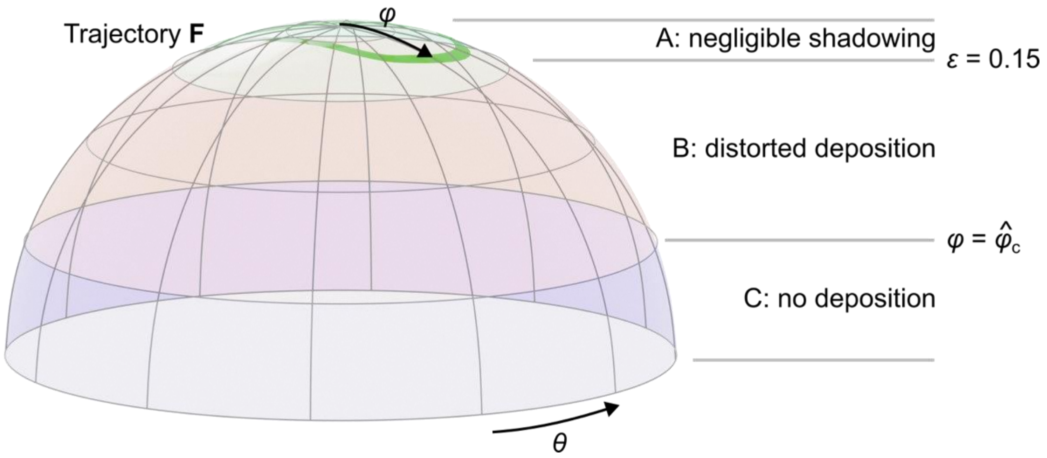

Supplementary Fig. 20. Operational regimes of MBHL for a certain stencil design. The parametric hemisphere is divided into 3 regimes categorized by the range of deposition angle φ. A) The self-shadowing effect is negligible, and the deposition process follows the convolution in Eq. (1) of the manuscript. B) The self-shadowing effect causes the deposited patterns to deviate from the convolution, and the deposition process is governed by Eq. (8) of the manuscript. C) No deposition occurs.

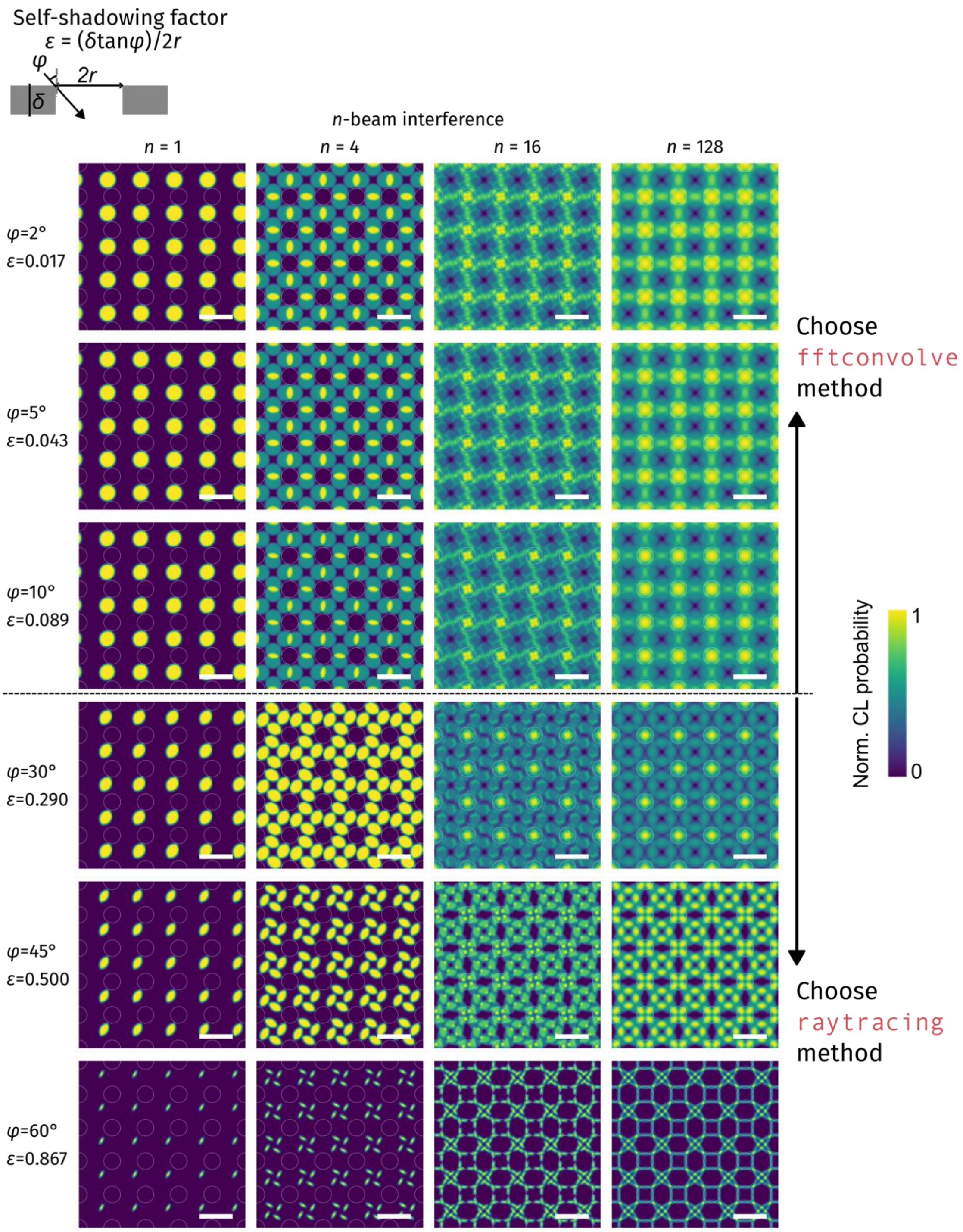

Supplementary Fig. 21. Evolution of MBHL pattern as function of polar angle φ in n-beam lithography. All simulation systems are periodic square lattice stencil with $r = 50$ nm, $L = 200$ nm, $\delta = 50$ nm, and a fixed $R = 200$ nm. Scale bars, 200 nm. The factor $\varepsilon = \frac{\delta \tan\varphi}{2r}$ determines the prominence of self-shadowing effect. For practical considerations, systems $\varepsilon < 0.1$ can be efficiently simulate using the fftconvolve method which ignores shadowing effect, while otherwise the full treatment in raytracing method is a must. Source data are provided as a Source Data file.

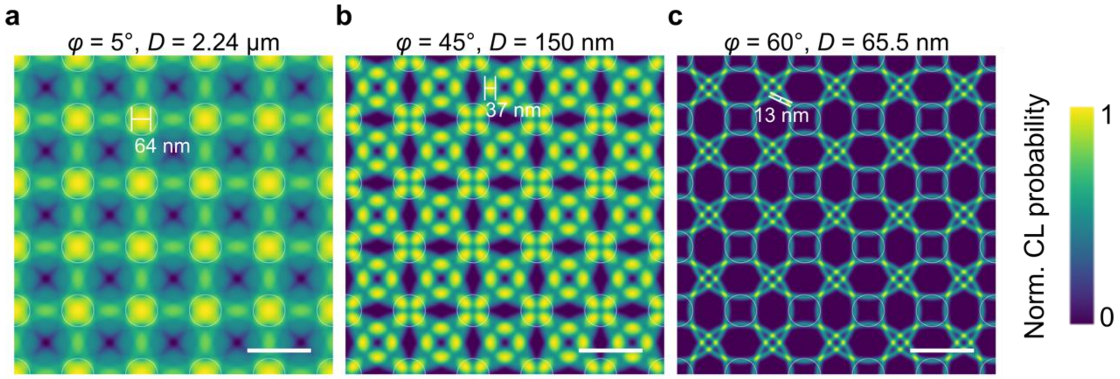

Supplementary Fig. 22. Finer structures formed under high-φ deposition conditions leveraging the self-shadowing effect. Examples were generated using the same stencil in Supplementary Fig. 21 under $n = \infty$ beam interference with: **a**, $\varphi = 5°, D = 2.24$μm. **b**, $\varphi = 45°, D = 150$ nm. **c**, $\varphi = 60°, D = 65.5$ nm. Scale bars: 200 nm. The locations of the nanoapertures are labeled in white circles. In all simulations, the radius of offset trajectory was constant at $R = 200$ nm. As φ increases, the self-shadowing effect intensifies, and the critical dimension of the MBHL patterns, measured using the most prominent surface features, decreases significantly, from 64 nm at $\varphi = 5°$ to only 13 nm at $\varphi = 60°$, well below the dimensions of the nanoapertures used. Notably, the small spacing ($D = 65.5$ nm) poses technical challenges that will be addressed in future studies. Source data are provided as a Source Data file.

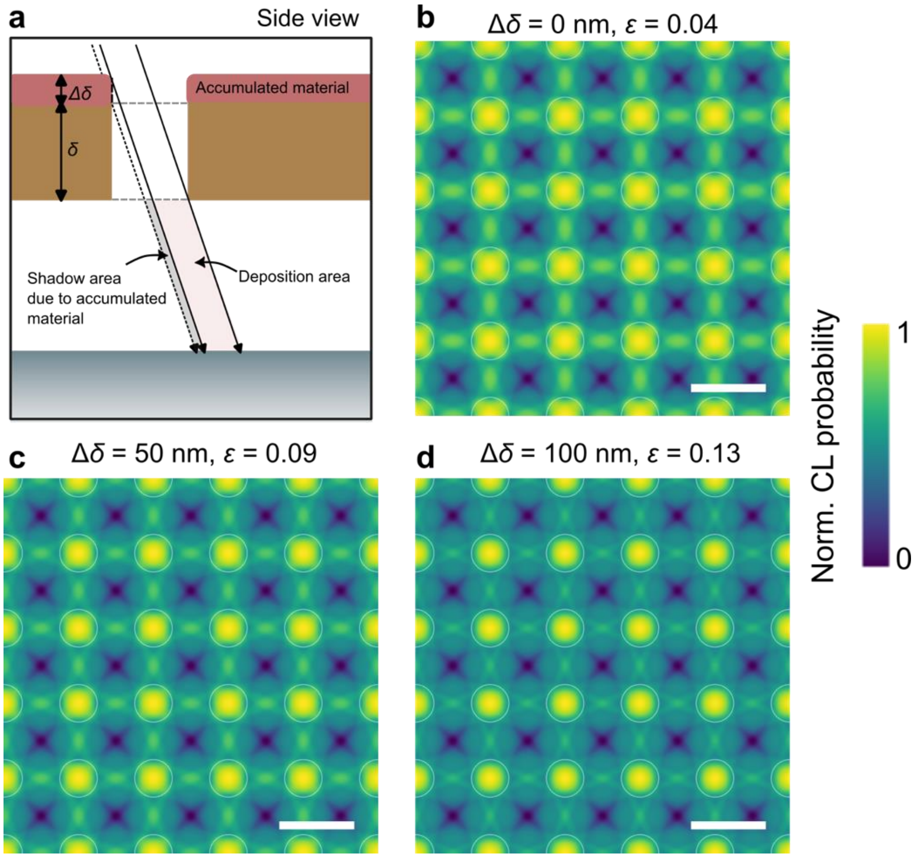

Supplementary Fig. 23. Simulating the effect of material accumulation on the top surface of the stencil. a, Schematic illustration showing the increase in membrane thickness $\Delta\delta$ due to material accumulation on the stencil, which leads to an increase in the shadowing factor ε. **b-d**, CL simulations for the probability of deposition $\mathbf{P}$ at different values of $\Delta\delta$ of 0 nm, 50 nm, and 100 nm, respectively. The MBHL parameters are identical to those used in Supplementary Fig. 19a. Scale bars, 200 nm. Source data are provided as a Source Data file.

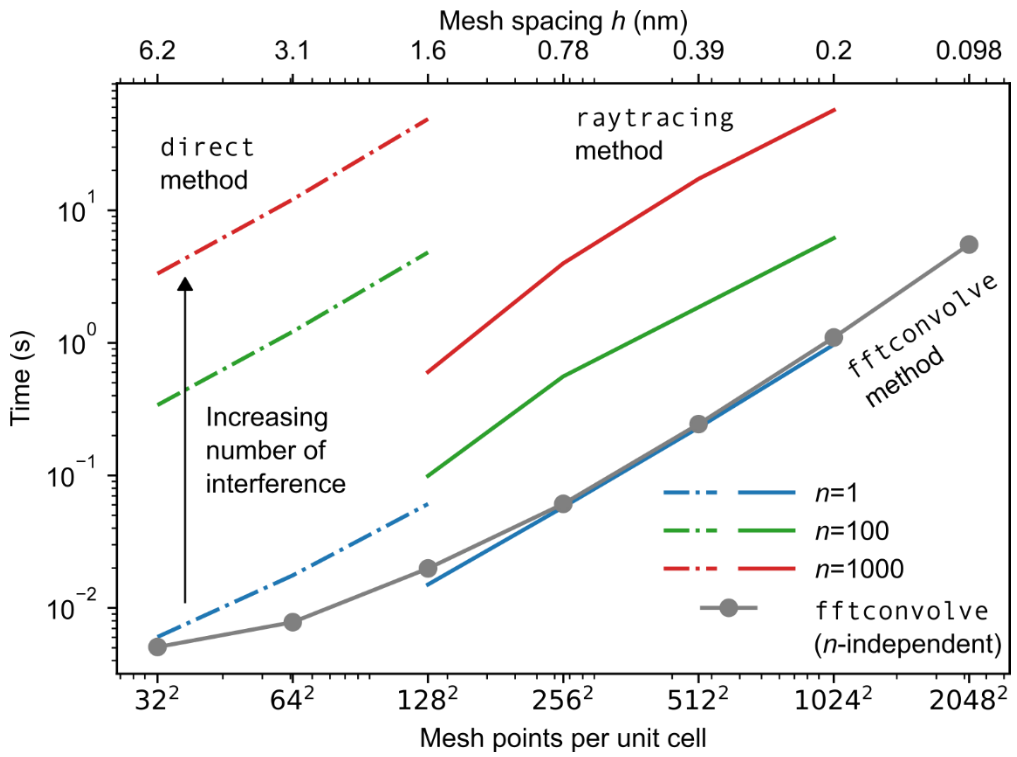

Supplementary Fig. 24. Performance of the numerical MBHL simulation measured by the simulation time as a function of mesh size per unit cell for various simulation approaches: **i)**. the direct approach, considering overlap between shapes formed by the nanoapertures and their self-shadow on the substrate plane. **ii).** the raytracing approach by calculating the contributions from incident particles according to main text Eq. (8), and **iii).** the fftconvolve approach according to main text Eq. (10). While the computation time for direct and raytracing methods linearly increase with the number of incident beams (n), the fftconvolve approach is independent of the trajectory size. The simulations were performed on a single-thread Apple-M1 CPU with periodic boundary conditions. Source data are provided as a Source Data file.

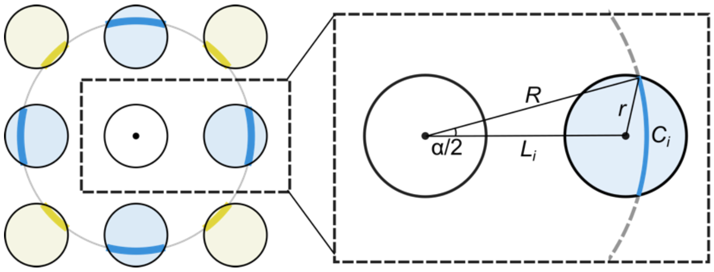

Supplementary Fig. 25. Details for an MBHL system with square-lattice nanopores, as depicted in main text Figure 2b.
